# The potential impact of primary headache disorders on stroke risk

**DOI:** 10.1186/s10194-016-0701-2

**Published:** 2016-12-01

**Authors:** Chia-Lin Tsai, Chung-Hsing Chou, Pei-Jung Lee, Jiu-Haw Yin, Shao-Yuan Chen, Chun-Chieh Lin, Yueh-Feng Sung, Fu-Chi Yang, Chi-Hsiang Chung, Wu-Chien Chien, Chia-Kuang Tsai, Jiunn-Tay Lee

**Affiliations:** 1 Department of Neurology, Tri-Service General Hospital, National Defense Medical Center, No.325, Sec. 2, Cheng-Kung Road, 11490 Taipei, Taiwan, Republic of China; 2 Graduate Institute of Medical Sciences, National Defense Medical Center, Taipei, Taiwan, ROC; 3 Departments of Nursing, Tri-Service General Hospital, National Defense Medical Center, Taipei, Taiwan, ROC; 4 Division of Neurology, Department of Medicine, Cheng Hsin General Hospital, Taipei, Taiwan, ROC; 5 Department of Neurology, Cardinal Tien Hospital, New Taipei City, Taiwan, ROC; 6 Department of Hyperbaric Medicine, Cardinal Tien Hospital, New Taipei City, Taiwan, ROC; 7 School of Medicine, Fu-Jen Catholic University, New Taipei City, Taiwan, ROC; 8 Taiwanese Injury Prevention and Safety Promotion Association, Taipei, Taiwan, ROC; 9 School of Public Health, National Defense Medical Center, Taipei, Taiwan, ROC; 10 Department of Medical Research, Tri-Service General Hospital, National Defense Medical Center, Taipei, Taiwan, ROC

**Keywords:** Tension-type headache, Migraine, Non-migrainous headache, Ischaemic stroke, Risk factor

## Abstract

**Background:**

Headache such as migraine is associated with stroke. Studies focused on primary headache disorders (PHDs) as a risk factor for stroke are limited. The purpose of this population-based cohort study was to explore whether patients with PHDs were at a high risk for developing stroke.

**Methods:**

A total of 1346 patients with PHDs were enrolled and compared with 5384 age-, gender- and co-morbidity-matched control cohorts. International Classification of Diseases, Clinical Modification codes were administered for the definition of PHDs, stroke, and stroke risk factors. Cox proportional-hazards regressions were performed for investigating hazard ratios (HR).

**Results:**

PHDs patients exhibited a 1.49 times (95% CI :1.15–1.98, *p* < 0.01) higher risk for developing ischaemic stroke compared with that of control cohorts. Both migraine (HR = 1.22, 95% CI :1.13–1.97, *p* < 0.05) and tension-type headache (HR = 2.29, 95% CI :1.22–2.80, *p* < 0.01) were associated with an increased risk of ischemic stroke. Females with PHDs were at greater risk of developing ischaemic stroke (HR = 1.49, 95% CI :1.13–1.90, *p* < 0.01) than those without PHDs. PHDs patient aged 45 to 64 years displayed significantly higher risk to develop ischaemic stroke (HR = 1.50, 95% CI: 1.11–2.10, *p* < 0.05) than the matched controls. The impact of PHDs on ischaemic stroke risk became gradually apparent by different following time intervals beyond 2 years after first diagnosis.

**Conclusion:**

PHDs is suggestive of an incremental risk for ischaemic stroke with gender-dependent, age-specific and time-dependent characteristics.

**Electronic supplementary material:**

The online version of this article (doi:10.1186/s10194-016-0701-2) contains supplementary material, which is available to authorized users.

## Background

Stroke is one of leading causative factors of permanent disability and mortality worldwide. It is considered as a preventive disorder and preventive interventions are of more considerable value compared with therapeutic approaches [1]. The crucial risk factors for stroke had been established, including non-modifiable (age, gender) and modifiable (hypertension, diabetes, dyslipidemia, smoking, and atrial fibrillation) [2]. Up to 40% patients with stroke have been reported to have medical conditions in addition to the traditional risk factors, which contribute to pathogenesis of stroke in certain populations [3, 4]. It is suggested that investigation of certain medical diseases or conditions to establish a full-scale account of risk factors for stroke achieve an appropriate prevention with great value.

Primary headache disorders (PHDs) include migraine, tension-type headache, cluster headache, and other primary headaches [5]. PHDs is known as recurrent or persistent pain of head without any clear underlying mechanism, which significantly impair patients’ life quality [6, 7]. Migraine is reported as a risk factor of stroke [8], whereas non- migraine headaches have received less notice regardless of high prevalence [9, 10]. Given the advantages of the Taiwan National Health Insurance program, the present study was intended to explore increased risks of stroke in patients with PHDs.

## Methods

### Data sources

The National Health Insurance (NHI) system, instituted in 1995, is a mandatory social insurance program that offers comprehensive health care coverage to all residents of Taiwan. This retrospective study was conducted using the data retrieved from Longitudinal Health Insurance Database (LHID 2005) released by Taiwan National Health Research Institute (NHRI) covering clinical data between 1997 and 2010. The LHID 2005 consisted of declared information, including records of inpatient, outpatient, and ambulatory care services of one million individuals insured, who randomly extracted from a database of more than 25 million cases. The International Classification of Diseases, Ninth Revision, Clinical Modification (ICD-9-CM) was employed for classification of diseases. According to Taiwan NHRI, there was no significant difference in the distribution of age and gender between the patients in the LHID 2005 and the original database [11]. The patient identity was blinded as a scrambled and anonymous number to protect the privacy of the insured people. Previous reports have shown the reliability of the diagnosis coding in the LHID [12, 13]. The study protocol was reviewed and approved by Institutional Review Board of Tri-Service General Hospital (TSGHIRB No.: 1-104-05-112).

### Study population

From 967,854 individual outpatient care data in the LHID from 2000 to 2005, patients with new diagnosis of PHD were identified and included. The subjects with a previous diagnosis of PHDs and stroke or with lacking identification of sex or individuals under 18 years of age were excluded. Patients were diagnosed with migraine (ICD-9-CM 346), tension-type headache (ICD-9-CM 307.81), and other headache syndromes (ICD-9-CM 339) for the first time from 2000 to 2005 (*N* = 1,346), as the study cohort. We randomly selected 5,384 individuals with a ratio of 4:1, to match the PHDs group according to age, gender and comorbidities include hypertension, diabetes mellitus, ischaemic heart diseases, hyperlipidaemia and atrial fibrillation. The diagnosis date of PHDs was considered as the index date. Each case was then followed up from the index date up to the date of stroke developed. For cases who did not suffer a stroke, the endpoint of tracking was defined as the last day of the study period (December 31, 2010) or the termination date of insurance.

### Definition of stroke subtypes and comorbidities

Subtypes of stroke were divided into ischaemic stroke (ICD-9-CM 433–437) and haemorrhagic (ICD-9-CM 430–432), respectively. Brain image such as computed tomography (CT) or magnetic resonance imaging (MRI) was used to verify the stroke diagnosis, which was not established if the patient was merely given stroke ICD codes without the procedure code of CT or MRI. Comorbidities known as major vascular risk factors including hypertension (ICD-9-CM 401–405), diabetes mellitus (ICD-9-CM 250), ischaemic heart diseases (ICD-9-CM 410–414), hyperlipidaemia (ICD-9-CM 272) and atrial fibrillation (ICD-9-CM 427.3) were identified prior to the index date based on the above ICD classification.

### Statistical analyses

Pearson chi-square test was administered to check the differences of categorical variables such as age groups, gender, hypertension, diabetes, ischaemic heart diseases, hyperlipidaemia and atrial fibrillation between the study and control cohort. After adjustment for the mentioned variables, Cox proportional hazard regressions were employed to evaluate the adjusted hazard ratio (HR) for the influence of PHDs on developing stroke. Kaplan-Meier analysis was performed to estimate the cumulative incidence of stroke for these two groups. Statistical Package for the Social Science version 22.0 (SPSS Inc., Chicago, IL, USA) was administered for all statistical analyses.

## Results

In this study, we identified and included 1,346 patients diagnosed with PHDs and 5,384 age- and gender-matched controls for comparison. The demographic features were presented in Table 1, showing that the distribution of age, sex, and comorbidities of the study group were similar to the control group. The results revealed that 100 (incidence: 162.62/10,000 person-years) all stroke events developed in the study cohort and 287 (incidence: 116/10,000 person-years) in the control group over a five-year observation period (Table 2). In the subgroup analysis, individuals with PHDs had a 1.49 times (95% CI: 1.15–1.98, *p* < 0.01) higher risk to develop ischaemic stroke, instead of haemorrhagic stroke, compared with the control group (Table 2).

**Table 1:** Baseline demographic status and co-morbidities compared between comparison and primary headache disorders (PHDs) group

Variable	PHDs cohort *N* = 1,346 (%)	Comparison cohort *N* = 5,384 (%)	*p*-value
Age, years (SD)^a^	47.38 (14.56)	46.74 (15.77)	0.183
<45	820 (60.92)	3,280 (60.92)	
45–64	404 (30.01)	1,616 (30.01)	
≥65	122 (9.07)	488 (9.07)	
Sex			0.999
Female	959 (71.25)	3,836 (71.25)	
Male	387 (28.75)	1,548 (28.75)	
Co-morbidities			
Hypertension	72 (5.35)	305 (5.66)	0.342
DM	25 (1.86)	101 (1.88)	0.732
IHD	14 (1.04)	53 (0.98)	0.476
Hyperlipidaemia	22 (1.63)	99 (1.84)	0.142
AF	1 (0.07)	7 (0.13)	0.503
Event (strokes) in the endpoint	100 (7.43)	287 (5.33)	0.002
Years of follow-up (SD)^a^	4.57 (1.22)	4.60 (1.20)	0.465

*Abbreviation*: *SD* standard deviation, *PHDs* primary headache disorders, *DM* diabetes mellitus, *IHD* ischaemic heart disease, *AF* atrial fibrillation

^a^Student’s *t*-test

**Table 2:** Incidence of stroke and stroke subtype and multivariate Cox proportional hazards regression analysis measured hazard ratio for study cohort

Variable	PHDs cohort			Comparison cohort			Adjusted HR (95% CI)
	Event	PYs	Rate	Event	PYs	Rate	
All strokes	100	6,149	162.62	287	24,742	116.00	1.40 (1.13–1.68)^**^
Haemorrhagic	4	6,149	6.50	30	24,742	12.12	0.55 (0.25–1.58)
Ischaemic	96	6,149	156.11	257	24,742	103.87	1.49 (1.15–1.98)^**^
<45 years							
All strokes	13	2,951	44.05	33	12,539	26.32	1.66 (0.79–2.97)
Haemorrhagic	1	2,951	3.39	7	12,539	5.58	0.63 (0.10–4.00)
Ischaemic	12	2,951	40.66	26	12,539	20.73	1.95 (0.97–3.74)
45–64 years							
All strokes	50	2,568	194.73	124	9,222	134.46	1.43 (1.03–2.00)^*^
Haemorrhagic	3	2,568	11.68	13	9,222	14.10	0.81 (0.15–2.77)
Ischaemic	47	2,568	183.04	111	9,222	120.36	1.50 (1.11–2.10)^*^
≧65 years							
All strokes	37	631	586.68	130	2,981	436.14	1.33 (0.91–1.91)
Haemorrhagic	0	631	0	10	2,981	33.55	0
Ischaemic	37	631	586.68	120	2,981	402.59	1.42 (0.99–2.08)
Male							
All strokes	35	1,730	202.36	101	6,918	146.00	1.37 (0.91–2.02)
Haemorrhagic	3	1,730	17.35	15	6,918	21.68	0.80 (0.25–2.70)
Ischaemic	32	1,730	185.02	86	6,918	124.32	1.46 (0.91–2.10)
Female							
All strokes	65	4,420	147.06	186	17,825	104.35	1.40 (1.04–1.80)^*^
Haemorrhagic	1	4,420	2.26	15	17,825	8.42	0.26 (0.02–2.00)
Ischaemic	64	4,420	144.80	171	17,825	95.93	1.49 (1.13–1.90)^**^

Model adjusted for age, sex, hypertension, DM, IHD, hyperlipidaemia, AF

*Abbreviation: PYs* person-years, *Rate* incidence rate, per 10,000 person-years, *PHDs* primary headache disorders, *DM* diabetes mellitus, *IHD* ischaemic heart disease, *AF* atrial fibrillation; HR: hazard ratio

*p < 0.05; **p < 0.01

To investigate whether PHDs is an age-dependent risk factor for ischaemic stroke, we stratified patients into 3 groups by age (<45, 45 to 64, and ≥65 years). The PHDs group had significantly greater risk for development of ischaemic stroke than the control cohort in the subgroup aged 45 to 64 (HR = 1.50, 95% CI: 1.11–2.10, *p* < 0.05). However, no significant difference was found between the PHDs group and matched controls with age <45 and ≥65 years. We also examined if PHDs is a gender-dependent risk factor for developing ischaemic stroke using the Cox regression analysis. The result of gender-specific analysis revealed that women with PHDs had higher risk to develop ischaemic stroke (HR = 1.49, 95% CI: 1.13–1.90, *p* < 0.01) than those without PHDs.

**Fig. 1:**
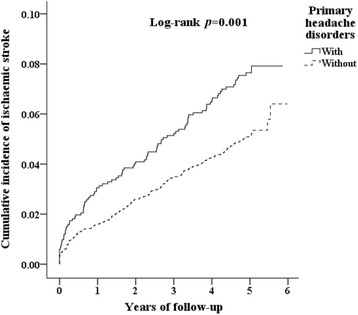
The cumulative incidence of ischemic stroke for the individual with and without primary headache disorders

Furthermore, we analyzed the incidence of stroke and stroke subtypes using multivariate Cox proportional hazards regression analysis according to time intervals. Our data revealed that patients with PHDs were likely to develop ischaemic stroke beyond 2 years after diagnosis (HR = 1.40, 95% CI: 1.00–1.90, *p* < 0.05) (Table 3). Significantly higher cumulative incidence of ischaemic stroke in the PHDs group than in the matched controls was observed by Kaplan-Meier analysis (Fig. 1).

**Table 3:** Incidence of stroke and stroke subtype and multivariate Cox proportional hazards regression analysis measured hazard ratio for study cohort by various time intervals

Variable	PHDs cohort			Comparison cohort			Adjusted HR (95% CI)
	Event	PYs	Rate	Event	PYs	Rate	
Follow <1 year							
All strokes	42	22	19,337.02	96	57	16,949.15	1.24 (0.85–1.87)
Haemorrhagic	2	22	920.81	12	57	2,118.64	0.49 (0.14–2.10)
Ischaemic	40	22	18,416.21	84	57	14,830.51	1.31 (0.91–1.88)
Follow ≧1, <2 years							
All strokes	13	43	3,046.64	55	176	3,122.52	1.00 (0.55–1.80)
Haemorrhagic	0	43	0	5	176	283.87	0
Ischaemic	13	43	3,046.64	50	176	2,838.65	1.04 (0.43–1.86)
Follow ≧2 years							
All strokes	45	6,085	73.95	136	24,510	55.49	1.33 (1.05–1.88)^*^
Haemorrhagic	2	6,085	3.29	13	24,510	5.30	0.63 (0.11–2.54)
Ischaemic	43	6,085	70.66	123	24,510	50.18	1.40 (1.00–1.90)^*^

Model adjusted for age, sex, hypertension, DM, IHD, hyperlipidaemia, AF

*Abbreviation: PYs* person-years, *Rate* incidence rate, per 10,000 person-years, *PHDs* primary headache disorders, *DM* diabetes mellitus, *IHD* ischaemic heart disease, *AF* atrial fibrillation

*p < 0.05

We next investigated the impact of the PHDs subsets on stroke. Distribution of PHDs was presented in Additional file 1: Table S1. The subgroup analysis showed that patients with either migraine or tension-type headache had a higher risk to develop ischaemic stroke (Table 4). Compared to the non-PHDs group, the risks of ischaemic stroke were 1.22 (95% CI: 1.13–1.97) and 2.29 (95% CI: 1.22–2.80) times higher in migraine and tension-type headache group, respectively.

**Table 4:** Incidence of stroke and stroke subtype and multivariate Cox proportional hazards regression analysis measured hazard ratio for study cohort by PHDs subtype

Variable	PHDs cohort			Comparison cohort			Adjusted HR (95% CI)
	Event	PYs	Rate	Event	PYs	Rate	
Total							
All strokes	100	6,149	162.62	287	24,742	116.00	1.40 (1.13–1.68)^**^
Haemorrhagic	4	6,149	6.50	30	24,742	12.12	0.55 (0.25–1.58)
Ischaemic	96	6,149	156.11	257	24,742	103.87	1.49 (1.15–1.98)^**^
Migraine							
All strokes	65	5,235	124.16	287	24,742	116.00	1.15 (1.04–1.69)*
Haemorrhagic	3	5,235	5.73	30	24,742	12.12	0.44 (0.21–1.57)
Ischaemic	62	5,235	118.43	257	24,742	103.87	1.22 (1.13–1.97)*
Tension-type headache							
All strokes	35	996	351.41	287	24,742	116.00	2.16 (1.25–2.77)**
Haemorrhagic	1	996	10.04	30	24,742	12.12	0.86 (0.23–1.64)
Ischaemic	34	996	341.37	257	24,742	103.87	2.29 (1.22–2.80)**

Model adjusted for age, sex, hypertension, DM, IHD, hyperlipidaemia, AF

*Abbreviation: PYs* person-years, *Rate* incidence rate, per 10,000 person-years, *PHDs* primary headache disorders, *DM* diabetes mellitus, *IHD* ischaemic heart disease, *AF* atrial fibrillation

*p < 0.05; **p < 0.01

## Discussion

In this nationwide, population-based cohort study, we found an incremental risk of ischaemic stroke in patients with PHDs. After adjusting for age, gender, and medical comorbidities, PHDs patients were more likely to develop ischemic stroke than matched controls. A subgroup analysis revealed that both migraine and tension-type headache were associated with an increased risk of ischemic stroke. Moreover, we showed that female PHDs patients had higher likelihood to develop ischaemic stroke. In addition, we found that the middle age group (aged 45 to 64 years) with PHDs was more susceptible to development of ischaemic stroke. It is worth noting that the impact of PHDs on developing ischaemic stroke was significant with an interval of at least 2 years follow-up and increased over time.

Accumulating evidence has highlighted the association of ischaemic stroke with previous migraine [8, 14–16]. A meta-analysis reviewing 13 case–control, 10 cohort, and two cross sectional studies has suggested that migraine is one of independent risk factors for ischaemic stroke [14]. However, the power of this meta-analysis is restricted chiefly by the case–control nature of many of the researches, with their innate susceptibility to recall bias. Non-migraine or chronic headache has been linked to an incremental risk of all strokes [17, 18], but the evidence is conflicting. Neither of these studies compared the association of chronic headache with stroke subtypes. In this longitudinal study using nation-based database, we observed that patients with either migraine or tension-type headache had an increased risk for development of ischaemic stroke.

Recently, the mechanisms underlying migraine as a possible risk factor of ischaemic stroke have been postulated. Speculated mechanisms consist mainly of vasospasm [19, 20], prolonged of cortical spreading depression [21], platelet hyperaggregability [22, 23], increased prothrombotic factors [24, 25], endothelial abnormalities [26, 27] and alteration of arterial function [28]. In addition, a recent systemic review and meta-analysis study [29] has reported an association between migraine with myocardial infarction, The study suggests that the association is more obvious and evident in women, which was in line with results of the present study.

Recently, studies have shown a subcortical white matter (WM) hyperintensity in migraine patients [30, 31]. An incremental risk of WM hyperintensity has been observed in patients with tension-type headache, [32] indicating that the link spreads to non-migrainous headaches. WM hyperintensity has been suggested as an incomplete process of ischemia with a result of arteriolosclerosis of small vessels in the cerebrum [33]. It is also considered as small vessel alterations leading dysfunction of blood–brain barrier, and subsequent chronic diffusion of macromolecules and fluids in the white matter; oxidative stress, dysfunction of endothelium and mechanisms involved in vascular regulation [34]. WM hyperintensity is related to an incremental risk for ischaemic stroke [35, 36]. As a result, subtle brain WM changes may lead to the higher risk for ischeamic stroke in PHDs patients. Further researches are necessary to verify these suppositions.

Compared to individuals without PHDs, patients with migraine had a null risk of haemorrhagic stroke in this study, which is consistent with the poor correlation between migraine and risk of haemorrhagic stroke in a previous study [37]. However, these findings differ from prior studies [15, 38] that indicated migraine was associated with an increased risk of haemorrhagic stroke. Moreover, migraine with aura rather than migraine without aura played a more important role in the overall increase of haemorrhagic stroke risk. Differences in methodological aspects and clinical settings might contribute to the discrepancies [39].

The prevalence of PHDs was higher for women than for men in our study as has been formerly reported [40]. This has been considered as a contribution of the effect of female hormones especially estrogen. Researches have shown that the risk for ischaemic stroke was increased in women who had migraine with aura and appeared to be exacerbated by smoking, oral contraceptive use and age <45 years [14]. Even though there are considerable methodological limitations in these studies [41]. Recent prospective cohort study [42] provided evidence that women with migraine were associated with an increased risk for major cardiovascular disease including myocardial infarction, stroke, and angina/coronary revascularization procedures, despite the mechanisms remain unclear. Similarly, the risk of ischaemic stroke was higher in the women with PHDs than in the control group in the present study.

Stroke is often considered an aging-related disorder, and the incidence of stroke grows with age among the general population. Researches show the risk of stroke approximately doubles each decade after age 55 [2]. However, strokes can develop at any age in reality. In this study, we found that the risk for development of ischaemic stroke was significantly greater in the PHDs group aged 45 to 64, instead of the elderly PHDs group (aged ≥65 years), suggesting that increasing age was not an absolute risk factor for developing ischaemic stroke in individuals with PHDs. Our results that there was no significant higher risk of ischaemic stroke in the PHDs group aged <45 years are in a disagreement with a previous study in which [14] migraine with aura was associated with an increased risk of ischemic stroke and the increase appeared to be exacerbated by smoking, oral contraceptive use and age <45 years. The differences might be explained by study designs and environmental factors involved. Furthermore, the impact of PHDs on ischaemic stroke risk was significant after 2 years of follow-up and increased over time, supporting the hypothesis of the chronic effect of PHDs on ischaemic stroke risk.

### Study limitations

There are certain limitations in this study. Firstly, medication likes hormonal contraceptives or life habits, such as smoking, drinking, customs of exercise and food that may affect the stroke risk did not include in the study. Secondly, our data from the huge database may comprise unrecognized recurrent individuals that may have suffered PHDs and stroke prior to1996 when NIH began to administer. Moreover, coding error might happen in the dataset. Thirdly, potential diagnosis biases and misclassification might exist due to the diagnoses of PHDs were ascertained retrospectively without further confirmations by headache experts. At last, our results only revealed an association instead of demonstrating straightforward relationship. Further researches are necessary to elaborate the mechanism underlying the association showed in this study.

## Conclusion

In this study, we provide subjective evidence supporting the hypothesis that PHDs patients are at relatively high risk for developing ischaemic stroke. The increase in the risk of ischaemic stroke associated with PHDs was mainly associated with migraine and tension-type headache rather than other primary headaches. PHDs are considered to be an age-specific, gender dependent and time-dependent risk factor of ischaemic stroke. These findings suggest that preventive strategies of ischaemic stroke might be ameliorated by being more careful with subgroups of patients, especially middle-aged (45- to 64-year) and female individuals with PHDs.

## Additional file

Additional file 1: Table S1. Distribution of PHDs. (DOC 55 kb)
