# Supplementary material for: The potential impact of primary headache disorders on stroke risk
Source: J Headache Pain. 2016 Dec 1;17(1):108. doi: 10.1186/s10194-016-0701-2 (PMC5130928; doi:10.1186/s10194-016-0701-2)
Supplement: Additional file 1: Table S1. — Distribution of PHDs. (DOC 55 kb) [file 10194_2016_701_MOESM1_ESM.doc]

Additional file 1: Table S1. Distribution of PHDs

|  | Total  N =1,346 | Migraine  N =1,116 | Tension-type headache  N =218 | Other headache syndromes  N =12 |
| --- | --- | --- | --- | --- |
| Age |  |  |  |  |
| <45 | 820 | 687 | 124 | 9 |
| 45-64 | 404 | 352 | 50 | 2 |
| ≥65 | 122 | 77 | 44 | 1 |
| Sex |  |  |  |  |
| Female | 959 | 813 | 141 | 5 |
| Male | 387 | 303 | 77 | 7 |

PHDs: Primary headache disorders
